# The association between different predictive biomarkers and mortality of COVID-19

**DOI:** 10.1186/s42269-022-00844-7

**Published:** 2022-05-31

**Authors:** Narges Ansari, Mina Jahangiri, Kimia Shirbandi, Mina Ebrahimi, Fakher Rahim

**Affiliations:** 1 Department of Internal Medicine, Isfahan Bone Metabolic Disorders Research Center, School of Medicine, Isfahan University of Medical Sciences, Isfahan, Iran; 2 Department of Biostatistics and Epidemiology, Faculty of Health, Ahvaz Jundishapur University of Medical Sciences, Ahvaz, Iran; 3 Ahvaz Jundishapur University of Medical Sciences, Ahvaz, Iran; 4 Thalassemia and Hemoglobinopathy Research Centre, Ahvaz Jundishapur University of Medical Sciences, Ahvaz, Iran; 5 Student Research Committee, Ahvaz Jundishapur University of Medical Sciences, Ahvaz, Iran

**Keywords:** COVID-19, Biomarker, HLA, Polymorphism

## Abstract

**Background:**

Immunocompromised individuals are expected to be more prone to severe diseases and, subsequently, death. Genetic disorders and polymorphisms in genes involved in the immune system, such as human leukocyte antigen (HLA), inflammatory cytokines, and killer-cell immunoglobulin-like receptors, can be involved in the immune system's response to various pathogens. In the current survey, the data were received from the world health organization, collected around the world.

**Results:**

Spearman's coefficient correlation test for evaluating the relationship between the Daily Death Rates (DDR) and immunological variables showed a statistically significant correlation between the DDR and all immunological variables except TNFa857T, TNFa863A IL2330G, and IL2166T (*P* < 0.001). Also, there was a statistically significant correlation between the DDR and some HLA markers.

**Conclusion:**

This meta-analysis study shows that predictive biomarkers and mortality of COVID-19 are associated with HLA markers. However, these results should be confirmed in a more structured agreement. It is worth noting that the design of new studies should consider potential diseases with poor prognoses because they are related to these immune genetic markers.

**Supplementary Information:**

The online version contains supplementary material available at 10.1186/s42269-022-00844-7.

## Background

The outbreak of new coronavirus (Novel Coronavirus-2019) or SARS-CoV-2 started in Wuhan, Hubei Province, China, in December 2019 and has spread rapidly to other parts of China the world (Zhu et al. 2020). Infection with the SARS-CoV-2 leading to COVID-19 disease usually occurs through saliva droplets released by coughing and sneezing in symptomatic patients, asymptomatic carriers, and before the onset of clinical symptoms (Singhal 2020). The current outbreak of COVID-19 disease has created a state of emergency and danger to public health internationally; therefore, governments have made new decisions to control and manage this crisis to cause minor damage to communities (Allain-Dupré et al. 2020). COVID-19 related mortality is usually measured by two parameters, including Case Fatality Rate (CFR) and Daily Death Rates (DDR) (Kim et al. 2021).

CFR is obtained by dividing the number of deaths by the number of confirmed cases of COVID-19, and DDR is calculated by dividing the number of deaths by the country's population (Onder et al. 2020; Eikenberry et al. 2020). Mortality rates vary widely from country to country, ranging from 0 to 31 percent and from 0 to 48 daily deaths per 10 million people. Mortality rates in Asia (lower than 1.3%) were lower than in Europe (1.8%) but reached 2.2% in the Americas, where Europeans are high (Ritchie et al. 2021).

Many factors can alter mortality rates in COVID-19 patients, resulting in changes in DDR and CFR levels (Cao et al. 2020). Patients with weakened immune systems are expected to be more prone to severe disease and death. Genetic disorders and the occurrence of polymorphisms in genes involved in the immune system, such as human leukocyte antigen (HLA), inflammatory cytokines, and killer-cell immunoglobulin-like receptors (KIR), can be involved in the immune system's response to various pathogens (Nguyen et al. 2020; Fara et al. 2020; Bernal et al. 2021).

This systematic review retrieved worldwide allele frequencies of WHO data and correlated the HLA, inflammatory cytokines, and KIR polymorphisms with DDR and CFR. Due to the extensive missing data, statistical analysis was performed in two ways: imputation and without imputation.

## Methods

### COVID-19 epidemiological statistics

In the present survey, we used a method that our research team recently published to retrieve data and estimate CFR and DDR. The data about total cases, total deaths, and total recovered cases, alongside total screening tests used to diagnose COVID-19, were collected since the beginnings of the COVID-19 pandemic for all countries from the world's most acceptable and accurate data repositories, World Health Organization (https://covid19.who.int/), Worldometer (Abdollahi et al. 2020), the Centers for Disease Control and Prevention, and the Morbidity and Mortality Weekly Report series (provided from Centers for Disease Control and Prevention) (Khafaie and Rahim 2020), consistent with the user's guide of data sources for patient registries.

The (i) CFR, which is obtained by dividing the number of deaths by the number of confirmed cases of COVID-19, (ii) Daily Death Rate (DDR), represented as the average number of deaths per day (since the first confirmed case) per ten million inhabitants were evaluated for each country.

### Statistical analysis

Descriptive statistics such as mean, standard deviation, median and interquartile range were computed for quantitative variables. The normality of data was evaluated using the Shapiro–Wilk test. Paired samples t-test was used to compare the CFR and DDR in May 2020-Nov, 2020 and Nov 2020-May 2021.

In addition, Spearman's coefficient correlation test was used to assess the association between quantitative variables and outcome variables (CFR and DDR). Cut-off values for interpretation of coefficient correlation are shown in Table 1 (Mukaka 2012). Data were measured and analyzed for each country, and CFR and DDR for countries with ≥ 1,000 cases (*n* = 90) are presented in Table 1.

**Table 1:**
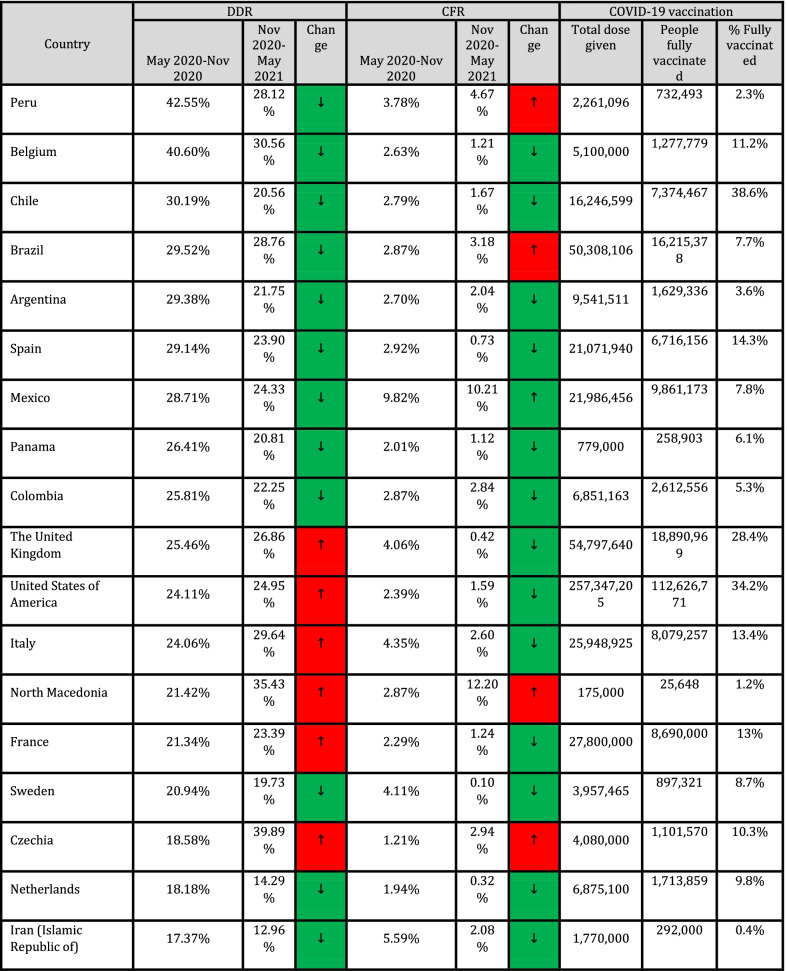

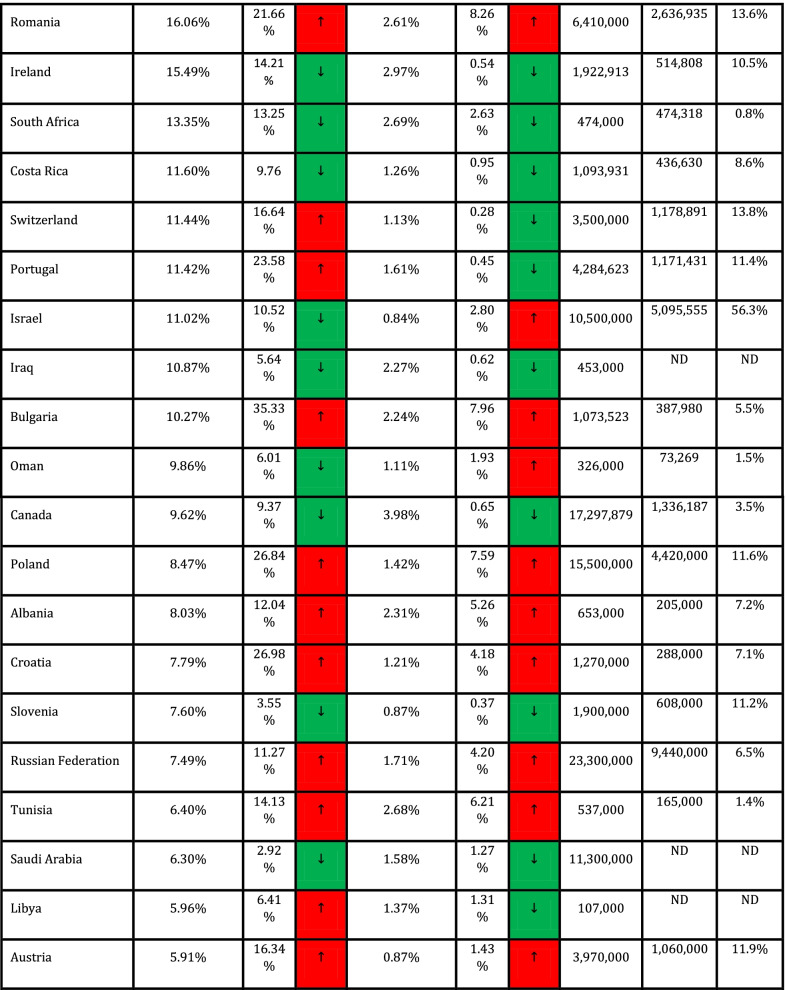

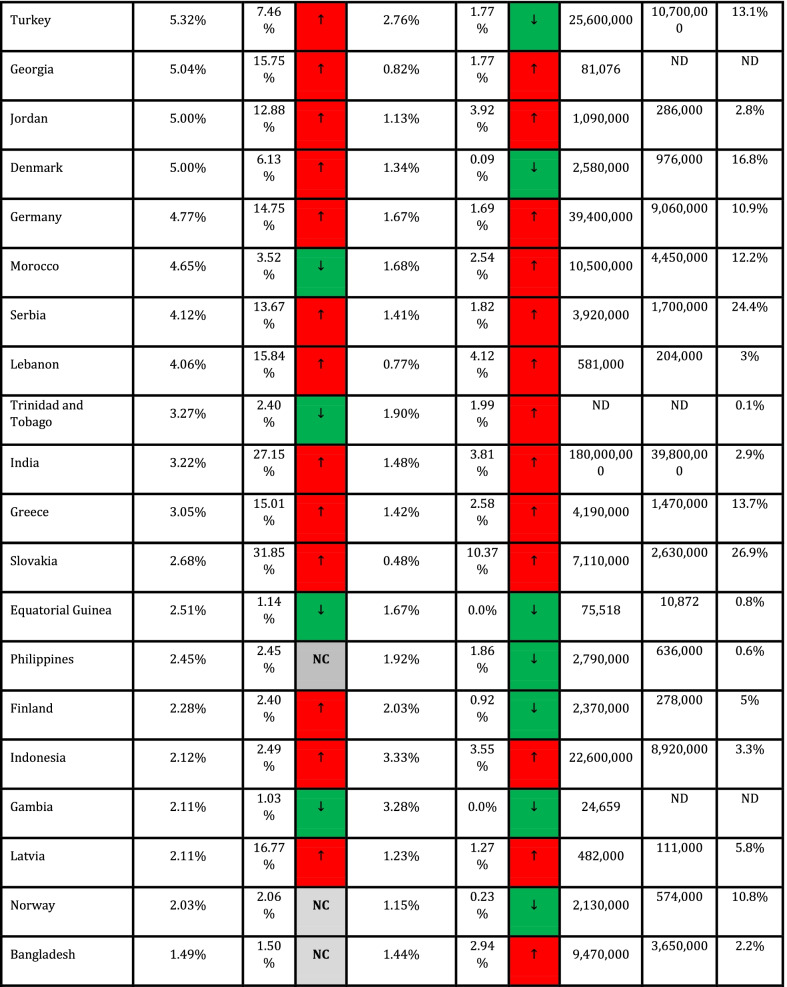

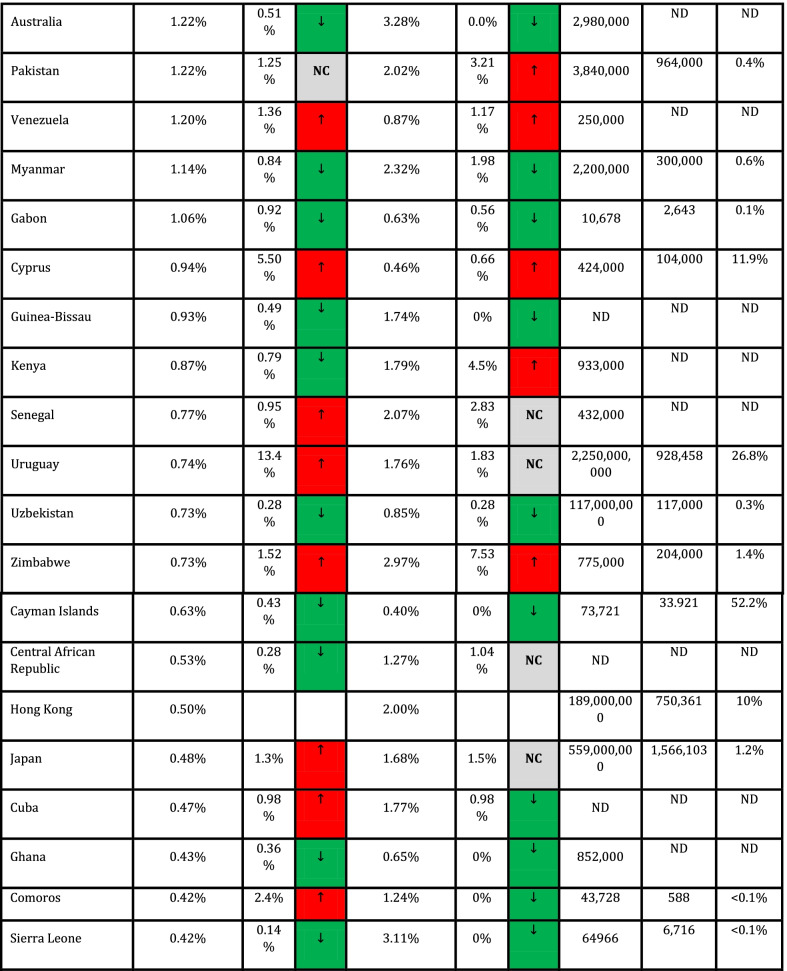

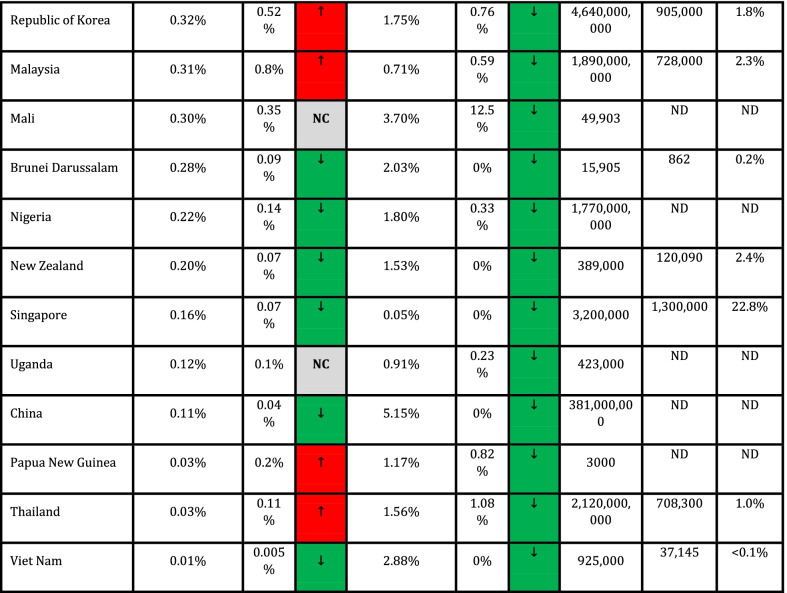
Correlation of CFR and DDR with COVID-19 vaccination frequencies across countries

Due to missing values in all quantitative variables (immunological and genetic variables and HLA markers) except CFR and DDR. Data analysis was done using MedCalc software and free statistical software R version 4.1.0. Package miss Forest was used to impute missing values. *P* < 0.05 was regarded as a significant statistical difference. NAM = North America; EUR = Europe; SAM = South and Central America; WAS = West Asia; CAS = Central Asia; OCE = Oceania; NAF = North Africa; SAS = South Asia; NEA = North-East Asia; SSA = Sub-Saharan Africa; SEA = South-East Asia.

## Results

**Fig. 1:**
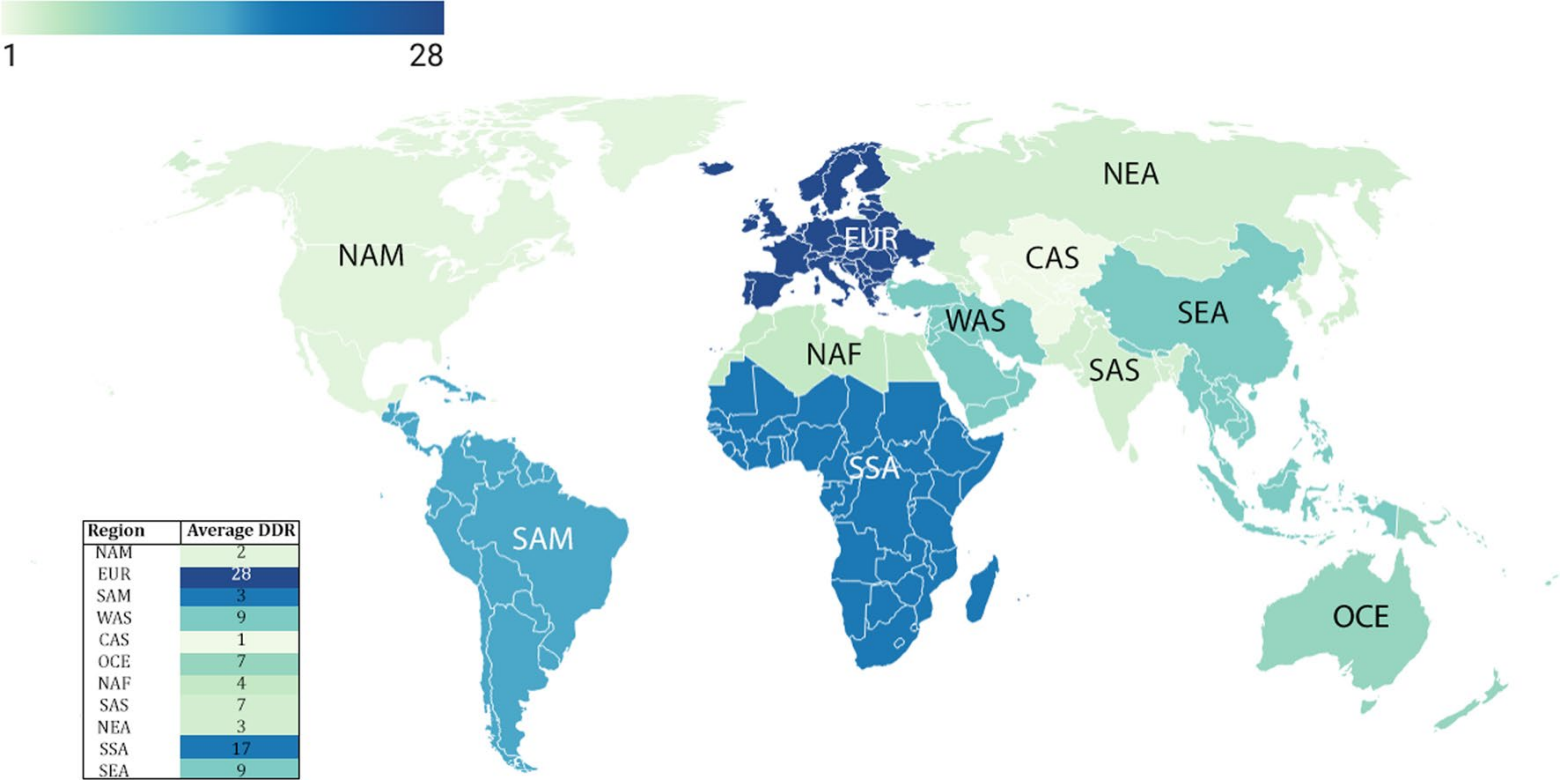
The frequency of the region

Collected data from 98 countries, which WHO reported, were analyzed. Descriptive statistics of DDR, CFR, and immunological and genetic variables are shown in Tables 1 and 2. In addition, HLA markers are shown in Additional file 1: Table S1. The frequency (%) of paises was equal to 1 (1%), and the region's frequency is shown in Fig. 1.

**Table 2:** Cut-off values for interpretation of coefficient correlation

Size of correlation	Interpretation
0.90–1.00 (− 0.90 to − 1.00)	Very high positive (negative) correlation
0.70–0.90 (− 0.70 to − 0.90)	High positive (negative) correlation
0.50–0.70 (− 0.50 to − 0.70)	Moderate positive (negative) correlation
0.30–0.50 (− 0.30 to − 0.50)	Low positive (negative) correlation
0.00–0.30 (− 0.00 to − 0.30)	Negligible correlation

The results show a statistically significant difference between the mean DDR in May 2020-Nov 2020 and Nov 2020-May 2021, and the mean DDR in Nov 2020-May 2021 is significantly higher than the mean DDR in May 2020-Nov 2020 (t (89) = − 2.98, *P* = 0.004). The percentage of DDR changes between these two time periods equals 28.32% (Table 3).

**Table 3:** Descriptive statistics of DDR, CFR, immunological and genetic variables

Variable		Minimum	Maximum	Mean	Standard. deviation	Median	Interquartile range
DDR		0	42.548	7.99	9.966	3.436	10.696
CFR		0	0.098	0.02	0.014	0.017	0.015
Immunological	IL6174C	0	0.5	0.216	0.144	0.244	0.261
	IL6565A	0	0.442	0.231	0.093	0.233	0.105
	TNFa238A	0.008	0.226	0.072	0.03	0.073	0.029
	TNFa308A	0.018	0.267	0.117	0.043	0.119	0.046
	TNFa1031C	0.103	0.436	0.213	0.033	0.3	0.02
	TNFa857T	0.01	0.282	0.122	0.036	0.124	0.029
	TNFa863A	0.065	0.331	0.152	0.029	0.15	0.02
	IFNg874T	0.072	0.579	0.348	0.108	0.373	0.178
	IFNg5644T	0.225	0.585	0.446	0.056	0.46	0.053
	IL101082G	0.024	0.549	0.4	0.103	0.357	0.081
	IL10592C	0.257	0.796	0.632	0.116	0.677	0.147
	IL10819C	0.249	0.8	0.634	0.115	0.7	0.138
	IL13962T	0.01	0.316	0.189	0.065	.203	0.091
	IL1511C	0.3	0.725	0.548	0.087	0.542	0.137
	IL2330G	0	0.568	0.315	0.111	0.4	0.1
	IL2166T	0.053	0.64	0.283	0.095	0.272	0.114
	IL121188A	0.456	0.832	0.682	0.077	0.7	0.088
Genetic	KirAAgenotype	1.5	55.275	29.665	8.93	28.029	9.235
	X.2DL2	13.45	71.90	50.761	10.453	53.116	13.249
	X.2DL3	56.50	99.70	87.92	6.27	88.66	5.166
	X.2DL5	28.40	78	54.615	8.255	55	9.086
	X.2DS1	1.4	64.60	37.253	10.967	39.441	9.568
	X.2DS2	13.80	71.70	49.221	10.495	50.95	12.775
	X.2DS3	10.30	52.20	29.258	7.991	28.886	10.33
	X.2DS5	0	83	34.567	9.484	33.62	7.963
	X.3DL1	75.50	100	93.848	4.824	94.516	4.662
	X.3DS1	0.7	62	34.687	12.77	37.548	12.311

Paired t-test also showed that there is no statistically significant difference between the mean CFR in May 2020-Nov 2020 and Nov 2020-May 2021 and the mean CFR in Nov 2020-May 2021 is higher than the mean CFR in May 2020-Nov 2020, and the percentage of CFR changes between these. The two-time intervals are equal to 7.69% (Table 3).

The results in Table 2 showed that there was a significantly high positive correlation between DDR in May 2020-Nov 2020 and Nov 2020-May 2021 (r = 0.87, *P* < 0.001). Spearman correlation coefficient also showed that there is a slight positive correlation between CFR in May 2020-Nov 2020 and Nov 2020-May 2021 (*r* = 0.16, *P* = 0.14) (Table 4).

**Table 4:** Comparison of DDR/CFR at May 2020-Nov 2020 and Nov 2020-May 2021

Variable	Time	Mean	Standard deviation	Mean of difference with 95% CI	Percentage of change (%)^*^	Statistics t (89)	*P* value^**^
DDR	May 2020-Nov 2020	8.37	10.23	− 2.37 (− 3.95, − 0.79)	28.32	− 2.98	0.004
	Nov 2020-May 2021	10.74	10.99				
CFR	May 2020-Nov 2020	2.08	1.35	− 0.16 (− 0.73, 0.40)	7.69	− 0.58	0.56
	Nov 2020-May 2021	2.24	2.75				

^**^: Paired samples t-test

:** ((Mean of after-Mean of before)/Mean of before) × 100

**Fig. 2:**
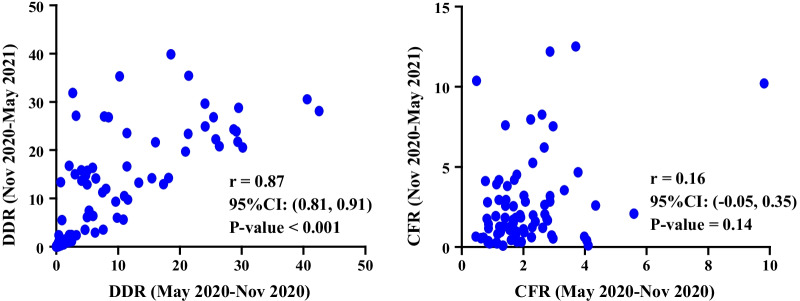
Scatter plots of DDR/CFR in May 2020-Nov, 2020 and Nov 2020-May, 2021

Figure 2 shows the distribution chart between DDR in May 2020-Nov 2020 and Nov 2020-May 2021 and the distribution chart between CFR in May 2020-Nov 2020 and Nov 2020-May 2021.

Spearman's coefficient correlation test for evaluating the relationship between the DDR and immunological variables showed a statistically significant correlation between the DDR and all immunological variables except TNFa857T, TNFa863A IL2330G, and IL2166T (*P* < 0.001). In addition, this test indicated a statistically significant correlation between DDR in all immunological variables except TNFa1031C and TNFa863A (Additional file 1: Tables S2 and S3). The findings in Table 5 showed a statistically significant correlation between the CFR and only immunological variables like TNFa1031C and TNFa863A (*P* < 0.05).

**Table 5:** Coefficient correlation between DDR/CFR at May 2020-Nov 2020 and Nov 2020-May 2021

Variable	Time	Coefficient correlation	%95 CI	*P* value^*^
DDR	May 2020-Nov 2020	0.87	(0.81,0.91)	< 0.001
	Nov 2020-May 2021			
CFR	May 2020-Nov 2020	0.16	(− 0.05,0.35)	0.14
	Nov 2020-May 2021			

^*^:Spearman rank correlation coefficient

Spearman's coefficient correlation indicated a statistically significant correlation between the DDR and all genetic variables except X.2DL5, X.2DS1, X.2DS5, X.3DL1, and X.3DS1 (*P* < 0.05). In addition, the results of the relationship between the CFR and genetic variables showed that these variables do have not a statistically significant correlation with CFR (Additional file 1: Tables S4 and S5).

The supplementary findings showed a statistically significant correlation between the DDR and all HLA markers except markers such as B21T, B1302, B1503, B3505, B3901, B4002, B4006, B4201, B4403, B4501, B4901, and B5201. Spearman's coefficient correlation test for evaluating the relationship between the CFR and HLA markers showed a statistically significant correlation between the CFR and HLA markers, including B1302 (*P* = 0.048), B3503 (*P* = 0.049), and B5001 (*P* = 0.033) (Additional file 1: Tables S6 and S7).

## Discussion

Many factors such as age, underlying disorder, gender, and genetics affect the strength of the immune response against pathogens (Zhang and Cao 2019; Viveiros et al. 2021). Genetic alterations in immune system molecules can be a central factor in the severity of COVID-19 and cause significant clinical changes and mortality (da Silveira et al. 2021; Li et al. 2020). Information on the relationship between immunogenetic factors and the prognosis of COVID-19 is scarce. Considering the vital role of the immune system and the importance of its changes in COVID-19 patients, in this study, we summarized the data related to genetic differences in immune factors (inflammatory cytokines, HLA, and KIR). We examined their relationship with mortality (CFR and DDR). Also, known denominators are affected by low test coverage, asymptomatic or mild cases (Rajgor et al. 2020). This study showed the CFR were 1.35 and 2.75, respectively, in 2020 and 2021. However, it is for COVID-19 lower than Severe Acute Respiratory Syndrome (SARS) (9.5%) and the Middle East respiratory syndrome (MERS) (34.4%) but higher than that of influenza (0.1%) (Rajgor et al. 2020).

### Case fatality rate (CFR) and daily death rates (DDR)

The CFR is obtained by dividing the number of deaths by the number of confirmed cases of COVID-19. Asymptomatic patients (carriers) or failure to perform diagnostic tests reduces this fraction's denominator. On the other hand, the confirmed cases in the denominator are recovered and infected people, so the number of infected patients who will die is unknown. In addition, many factors, such as economic, political, and social conditions, also affect the amount of denominator. The DDR is calculated by dividing the number of deaths by the country's population and is used in countries with limited ability to perform tests. The data analysis shows that CAS (1%) with minimum and EUR (28%) with maximum average DDR.

Restrictions on testing in different countries have a lower impact on the number of deaths than in some cases. Therefore, DDR seems to be more suitable for COVID-19 than CFR. The studied parameters had the most significant relationship with DDR in the present study.

### Cytokines

The levels of TNFa, IL-6, IL-1b, IFNg, IL-2, and IL-10 have been reported as inflammatory cytokines in COVID-19 (Pedersen and Ho 2020). The cytokine polymorphisms with the allele frequencies Spearman's correlation with CFR and DDR estimates presents in (Additional file 1: Tables S2 and S3). However, the lack of significance after correction for multiple tests, the trend for positive correlation of inhibitory genotype AA and KIR2DL3 with DDR has been reported in the literature (Wang and Xia 2004), suggesting that low NK cell activity may be relevant in SARS. It is interesting to note that, despite the lack of significance after correction for multiple tests, the trend for positive correlation of inhibitory genotype AA and KIR2DL3 with DDR has been reported in the literature. As a result, there is no obvious pattern in HLA-B ligands. NK cells also react to and generate cytokines such as IL-12 and IL-2, as well as IFNg, TNFa, and IL-6 (Semino and Rubartelli 2010), with all these cytokines being amplified in the COVID-19 cytokine storm. Furthermore, TNFA is near to the HLA-B and HLA-C genes, as well as the HLA-B, HLA-C, and TNFA SN determination.

### Genetics

There was a strong relation between DDR and genetic variables. However, there was no correlation between CFR and genetic variables (Additional file 1: Tables S4 and S5).

In addition, some studies have shown that COVID-19 is related to genetic factors, mainly related to the immune response, such as HLA class I genes (Wang et al. 2020). The ongoing genome-wide association studies (GWAS) initiatives may reveal additional key genetic markers (Ovsyannikova et al. 2020). Therefore, the trend related to related immune genes indirectly confirms that the immune status will result from important participants in the background of COVID-19.

### HLA

The results showed that the association of different HLA markers with DDR is stronger than CFR. The Spearman's correlation coefficient shows that some HLA markers with DDR have shown a significance level. However, just B1302, B3503, and B5001 HLA markers with CFR have shown a significant level (Additional file 1: Tables S6 and S7).

HLAB* 07:03 (Ng et al. 2004) and HLA-B* 46:01 (Lin et al. 2003) were the first predisposing alleles suggested for SARS-CoV. However, these associations cannot be confirmed later (Xiong et al. 2008; Ng 1 et al. 2010; Leite et al. 2021).

HLA-B displays the strongest selective signal (Prugnolle et al. 2005) and associations with infectious diseases. In this context (Blackwell et al. 2009), along with evidence that the HLA-B molecule's affinity to SARS-CoV-2 epitopes plays a role in the infection.

## Conclusions

This study set out to determine the value of the predictive biomarker in COVID-19 mortality. Specific HLA and cytokine polymorphism emerge as a reliable prediction of DDR. Whereas just HLA B1302, B3503, and B 5001 were associated with CFR. Definitive diagnosis of patients with COVID-19 is the main challenge. To achieve this, minimize the interfering factors, including asymptomatic patients, subjects with mild symptoms, and COVID-19 misdiagnosis. On the other hand, it is worth noting that the design of new studies should consider potential diseases with poor prognoses because they are related to these immune genetic markers.

## Supplementary Information

**Additional file 1:** Further analysis between biomarkers and CFR and DDR.

## Data Availability

The authors are willing to share all data used in this study upon a reasonable request from the corresponding author.

## Abbreviations

HLA: Human leukocyte antigen
KIR: Killer-cell immunoglobulin-like receptors
WHO: World health organization
DDR: Daily death rates
Novel Coronavirus-2019: New coronavirus
CFR: Case fatality rate
