# Supplementary material for: The association between different predictive biomarkers and mortality of COVID-19
Source: Bull Natl Res Cent. 2022 May 31;46(1):158. doi: 10.1186/s42269-022-00844-7 (PMC9152825; doi:10.1186/s42269-022-00844-7)
Supplement: Supplementary file 1 — Additional file 1: Further analysis between biomarkers and CFR and DDR. [file 42269_2022_844_MOESM1_ESM.docx]

## **Table 1.** Descriptive statistics of HLA markers.

| **Variable** | **Minimum** | **Maximum** | **Mean** | **Standard. Deviation** | **Median** | **Interquartile range** |
| --- | --- | --- | --- | --- | --- | --- |
| **B21M** | 0 | 0.402 | 0.167 | 0.071 | 0.171 | 0.056 |
| **B21T** | 0.269 | 0.731 | 0.588 | 0.078 | 0.593 | 0.056 |
| **B0702** | 0 | 0.188 | 0.051 | 0.037 | 0.046 | 0.026 |
| **B0801** | 0 | 0.182 | 0.046 | 0.035 | 0.047 | 0.046 |
| **B1301** | 0 | 0.187 | 0.014 | 0.029 | 0.002 | 0.015 |
| **B1302** | 0 | 0.056 | 0.015 | 0.012 | 0.013 | 0.013 |
| **B1402** | 0 | 0.064 | 0.019 | 0.014 | 0.02 | 0.014 |
| **B1501** | 0 | 0.145 | 0.028 | 0.028 | 0.021 | 0.028 |
| **B1502** | 0 | 0.22 | 0.014 | 0.036 | 0.0004 | 0.008 |
| **B1503** | 0 | 0.108 | 0.017 | 0.023 | 0.008 | 0.02 |
| **B1801** | 0 | 0.133 | 0.039 | 0.024 | 0.038 | 0.031 |
| **B2705** | 0 | 0.152 | 0.013 | 0.019 | 0.008 | 0.016 |
| **B3501** | 0 | 0.144 | 0.052 | 0.306 | 0.052 | 0.027 |
| **B3503** | 0 | 0.111 | 0.018 | 0.017 | 0.014 | 0.019 |
| **B3505** | 0 | 0.515 | 0.012 | 0.053 | 0.001 | 0.006 |
| **B3801** | 0 | 0.055 | 0.013 | 0.013 | 0.009 | 0.016 |
| **B3901** | 0 | 0.114 | 0.014 | 0.018 | 0.01 | 0.01 |
| **B4001** | 0 | 0.228 | 0.033 | 0.039 | 0.022 | 0.033 |
| **B4002** | 0 | 0.398 | 0.025 | 0.047 | 0.013 | 0.012 |
| **B4006** | 0 | 0.121 | 0.01 | 0.018 | 0.004 | 0.012 |
| **B4201** | 0 | 0.138 | 0.012 | 0.023 | 0.003 | 0.01 |
| **B4402** | 0 | 0.126 | 0.028 | 0.026 | 0.025 | 0.041 |
| **B4403** | 0 | 0.11 | 0.034 | 0.022 | 0.036 | 0.021 |
| **B4501** | 0 | 0.109 | 0.015 | 0.02 | 0.008 | 0.018 |
| **B4601** | 0 | 0.163 | 0.009 | 0.025 | 0.0002 | 0.005 |
| **B4801** | 0 | 0.124 | 0.01 | 0.022 | 0.002 | 0.012 |
| **B4901** | 0 | 0.054 | 0.016 | 0.012 | 0.017 | 0.02 |
| **B5001** | 0 | 0.166 | 0.02 | 0.027 | 0.014 | 0.019 |
| **B5101** | 0 | 0.209 | 0.062 | 0.039 | 0.06 | 0.048 |
| **B5201** | 0 | 0.122 | 0.023 | 0.021 | 0.02 | 0.018 |
| **B5301** | 0 | 0.192 | 0.024 | 0.034 | 0.013 | 0.021 |
| **B5601** | 0 | 0.22 | 0.012 | 0.031 | 0.005 | 0.01 |
| **B5701** | 0 | 0.071 | 0.016 | 0.013 | 0.013 | 0.019 |
| **B5801** | 0 | 0.083 | 0.03 | 0.021 | 0.028 | 0.028 |

## **Table 2.** Relation between DDR and immunological variables (SCC: Spearman's Correlation Coefficient and CI: Confidence Interval).

| **Variable** | **SCC** | **95% CI** | **P-value** |
| --- | --- | --- | --- |
| **IL6174C** | 0.609 | (0.467, 0.72) | < 0.001 |
| **IL6565A** | 0.647 | (0.515, 0.75) | < 0.001 |
| **TNFa238A** | 0.332 | (0.143, 0.497) | < 0.001 |
| **TNFa308A** | 0.469 | (0.288, 0.603) | < 0.001 |
| **TNFa1031C** | - 0.219 | (-0.4, -0.022) | 0.03 |
| **TNFa857T** | 0.14 | (-0.06, 0.329) | 0.17 |
| **TNFa863A** | - 0.182 | (-0.367, 0.017) | 0.072 |
| **IFNg874T** | 0.571 | (0.42, 0.691) | < 0.001 |
| **IFNg5644T** | 0.454 | (0.281, 0.599) | < 0.001 |
| **IL101082G** | 0.612 | (0.471, 0.723) | < 0.001 |
| **IL10592C** | 0.642 | (0.509, 0.746) | < 0.001 |
| **IL10819C** | 0.63 | (0.494, 0.737) | < 0.001 |
| **IL13962T** | 0.538 | (0.38, 0.665) | < 0.001 |
| **IL1511C** | 0.501 | (0.336, 0.636) | < 0.001 |
| **IL2330G** | 0.066 | (-0.134, 0.261) | 0.519 |
| **IL2166T** | 0.17 | (-0.029, 0.356) | 0.094 |
| **IL121188A** | 0.612 | (0.471, 0.723) | < 0.001 |

## **Table 3.** Relation between CFR and immunological variables (SCC: Spearman's Correlation Coefficient and CI: Confidence Interval).

| **Variable** | **SCC** | **95% CI** | **P-value** |
| --- | --- | --- | --- |
| **IL6174C** | - 0.049 | (-0.245, 0.151) | 0.63 |
| **IL6565A** | 0.013 | (-0.186, 0.211) | 0.899 |
| **TNFa238A** | 0.165 | (-0.034, 0.352) | 0.104 |
| **TNFa308A** | 0.145 | (-0.055, 0.334) | 0.154 |
| **TNFa1031C** | - 0.361 | (-0.522, -0.175) | < 0.001 |
| **TNFa857T** | - 0.123 | (-0.313, 0.078) | 0.229 |
| **TNFa863A** | - 0.251 | (-0.429, -0.056) | 0.012 |
| **IFNg874T** | - 0.107 | (-0.299, -0.093) | 0.293 |
| **IFNg5644T** | - 0.195 | (-0.379, -0.004) | 0.054 |
| **IL101082G** | 0.055 | (-0.145, -0.251) | 0.588 |
| **IL10592C** | - 0.039 | (-0.236, 0.16) | 0.699 |
| **IL10819C** | - 0.054 | (-0.249, 0.146) | 0.6 |
| **IL13962T** | - 0.112 | (-0.304, 0.088) | 0.271 |
| **IL1511C** | - 0.104 | (-0.296, 0.096) | 0.308 |
| **IL2330G** | - 0.158 | (-0.346, 0.042) | 0.12 |
| **IL2166T** | 0.083 | (-0.117, 0.277) | 0.416 |
| **IL121188A** | - 0.011 | (-0.209, 0.188) | 0.916 |

## **Table 4.** Relation between DDR and genetic variables (SCC: Spearman's Correlation Coefficient and CI: Confidence Interval).

| **Variable** | **SCC** | **95% CI** | **P-value** |
| --- | --- | --- | --- |
| **KirAAgenotype** | - 0.32 | (-0.488, -0.013) | 0.001 |
| **X.2DL2** | 0.239 | (0.042, 0.417) | 0.018 |
| **X.2DL3** | - 0.226 | (-0.406, -0.029) | 0.025 |
| **X.2DL5** | 0.169 | (-0.03, 0.355) | 0.096 |
| **X.2DS1** | 0.079 | (-0.121, 0.273) | 0.438 |
| **X.2DS2** | 0.333 | (0.144, 0.498) | < 0.001 |
| **X.2DS3** | 0.227 | (0.03, 0.407) | 0.024 |
| **X.2DS5** | - 0.039 | (-0.235, 0.161) | 0.704 |
| **X.3DL1** | - 0.049 | (-0.245, 0.150) | 0.629 |
| **X.3DS1** | 0.162 | (-0.037, 0.349) | 0.11 |

## **Table 5.** Relation between CFR and genetic variables (SCC: Spearman's Correlation Coefficient and CI: Confidence Interval).

| **Variable** | **SCC** | **95% CI** | **P-value** |
| --- | --- | --- | --- |
| **KirAAgenotype** | 0.015 | (-0.184, 0.213) | 0.881 |
| **X.2DL2** | - 0.114 | (-0.306, 0.086) | 0.262 |
| **X.2DL3** | 0.159 | (-0.041, 0.346) | 0.119 |
| **X.2DL5** | - 0.016 | (-0.214, 0.183) | 0.874 |
| **X.2DS1** | 0.007 | (-0.191, 0.206) | 0.942 |
| **X.2DS2** | - 0.156 | (-0.344, 0.044) | 0.125 |
| **X.2DS3** | - 0.105 | (-0.297, 0.095) | 0.303 |
| **X.2DS5** | 0.103 | (-0.098, 0.295) | 0.315 |
| **X.3DL1** | - 0.007 | (-0.205, 0.192) | 0.947 |
| **X.3DS1** | 0.148 | (-0.052, 0.337) | 0.146 |

## **Table 6.** Relation between DDR and HLA markers (SCC: Spearman's Correlation Coefficient and CI: Confidence Interval).

| **Variable** | **SCC** | **95% CI** | **P-value** |
| --- | --- | --- | --- |
| **B21M** | 0.383 | (0.2, 0.54) | < 0.001 |
| **B21T** | - 0.104 | (-0.296, 0.097) | 0.309 |
| **B0702** | 0.365 | (0.18, 0.526) | < 0.001 |
| **B0801** | 0.501 | (0.336, 0.636) | < 0.001 |
| **B1301** | - 0.436 | (-0.584, -0.261) | < 0.001 |
| **B1302** | 0.093 | (-0.108, 0.286) | 0.364 |
| **B1402** | 0.401 | (0.221, 0.556) | < 0.001 |
| **B1501** | 0.253 | (0.058, 0.43) | 0.012 |
| **B1502** | - 0.27 | (-0.445, -0.076) | 0.007 |
| **B1503** | - 0.046 | (-0.242, 0.154) | 0.654 |
| **B1801** | 0.238 | (0.042, 0.417) | 0.018 |
| **B2705** | 0.536 | (0.378, 0.664) | < 0.001 |
| **B3501** | 0.379 | (0.195, 0.537) | < 0.001 |
| **B3503** | 0.365 | (0.179, 0.525) | < 0.001 |
| **B3505** | - 0.194 | (-0.377, 0.005) | 0.056 |
| **B3801** | 0.505 | (0.341, 0.64) | < 0.001 |
| **B3901** | 0.07 | (-0.13, 0.265) | 0.495 |
| **B4001** | - 0.256 | (-0.433, -0.061) | 0.011 |
| **B4002** | 0.033 | (-0.167, 0.23) | 0.749 |
| **B4006** | - 0.182 | (-0.367, 0.018) | 0.074 |
| **B4201** | - 0.053 | (-0.249, 0.147) | 0.604 |
| **B4402** | 0.476 | (0.307, 0.617) | < 0.001 |
| **B4403** | 0.155 | (-0.044, 0.343) | 0.126 |
| **B4501** | - 0.017 | (-0.215, 0.182) | 0.869 |
| **B4601** | - 0.381 | (-0.539, -0.197) | < 0.001 |
| **B4801** | - 0.245 | (-0.423, -0.049) | 0.015 |
| **B4901** | 0.144 | (-0.056, 0.333) | 0.158 |
| **B5001** | 0.242 | (0.046, 0.42) | 0.016 |
| **B5101** | 0.4 | (0.219, 0.555) | < 0.001 |
| **B5201** | - 0.055 | (-0.251, 0.145) | 0.588 |
| **B5301** | -0.042 | (-0.238, 0.158) | 0.682 |
| **B5601** | -0.385 | (-0.542, -0.202) | < 0.001 |
| **B5701** | 0.511 | (0.348, 0.644) | < 0.001 |
| **B5801** | - 0.438 | (-0.586, -0.262) | < 0.001 |

## **Table 7.** Relation between CFR and HLA markers (SCC: Spearman's Correlation Coefficient and CI: Confidence Interval).

| **Variable** | **SCC** | **95% CI** | **P-value** |
| --- | --- | --- | --- |
| **B21M** | 0.011 | (-0.188, 0.209) | 0.912 |
| **B21T** | - 0.084 | (-0.278, 0.116) | 0.409 |
| **B0702** | 0.005 | (-0.193, 0.204) | 0.958 |
| **B0801** | 0.028 | (-0.171, 0.225) | 0.782 |
| **B1301** | - 0.012 | (-0.21, 0.187) | 0.908 |
| **B1302** | - 0.201 | (-0.384, -0.003) | 0.048 |
| **B1402** | 0.054 | (-0.146, 0.25) | 0.598 |
| **B1501** | 0.022 | (-0.177, 0.22) | 0.828 |
| **B1502** | 0.112 | (-0.088, 0.304) | 0.273 |
| **B1503** | 0.014 | (-0.185, 0.212) | 0.894 |
| **B1801** | - 0.193 | (-0.377, 0.006) | 0.057 |
| **B2705** | 0.074 | (-0.126, 0.269) | 0.466 |
| **B3501** | 0.043 | (-0.157, 0.239) | 0.676 |
| **B3503** | - 0.199 | (-0.382, -0.001) | 0.049 |
| **B3505** | 0.084 | (-0.116, 0.278) | 0.408 |
| **B3801** | -0.108 | (-0.3, 0.092) | 0.29 |
| **B3901** | 0.092 | (-0.108, 0.286) | 0.365 |
| **B4001** | 0.041 | (-0.158, 0.238) | 0.686 |
| **B4002** | - 0.1 | (-0.293, 0.1) | 0.326 |
| **B4006** | 0.006 | (-0.192, 0.204) | 0.952 |
| **B4201** | - 0.015 | (-0.213, 0.184) | 0.881 |
| **B4402** | - 0.023 | (-0.221, 0.176) | 0.82 |
| **B4403** | 0.036 | (-0.164, 0.233) | 0.726 |
| **B4501** | 0.062 | (-0.138, 0.257) | 0.546 |
| **B4601** | - 0.068 | (-0.263, 0.132) | 0.505 |
| **B4801** | 0.072 | (-0.128, 0.267) | 0.48 |
| **B4901** | -0.123 | (-0.314, 0.077) | 0.226 |
| **B5001** | - 0.216 | (-0.397, -0.018) | 0.033 |
| **B5101** | - 0.144 | (-0.333, 0.056) | 0.157 |
| **B5201** | - 0.057 | (-0.253, 0.143) | 0.575 |
| **B5301** | - 0.052 | (-0.248, 0.148) | 0.612 |
| **B5601** | - 0.084 | (-0.278, 0.116) | 0.409 |
| **B5701** | 0.063 | (-0.137, 0.258) | 0.539 |
| **B5801** | - 0.069 | (-0.264, 0.132) | 0.502 |
